# Assessing the osseointegration potential of a strontium releasing nanostructured titanium oxide surface: A biomechanical study in the rabbit tibia plateau model

**DOI:** 10.1002/cre2.812

**Published:** 2023-12-03

**Authors:** Sila Cagri Isler, Benjamin Bellon, Morten Foss, Benjamin Pippenger, Andreas Stavropoulos, Ole Zoffmann Andersen

**Affiliations:** ^1^ Department of Periodontology, School of Dental Medicine University of Bern Bern Switzerland; ^2^ Department of Periodontology, Faculty of Dentistry Gazi University Ankara Turkey; ^3^ Preclinical & Translational Research Institut Straumann AG Basel Switzerland; ^4^ Department of Periodontology, Faculty of Dentistry University of Zurich Zurich Switzerland; ^5^ iNANO and Department of Physics and Astronomy Science and Technology Aarhus Denmark; ^6^ Department of Periodontology, Faculty of Odontology Malmö University Malmö Sweden; ^7^ Division of Conservative Dentistry and Periodontology, University Clinic of Dentistry Medical University of Vienna Vienna Austria

**Keywords:** osseointegration, preclinical in vivo, SLA, Strontium

## Abstract

**Objectives:**

To investigate the impact of a Ti‐Sr‐O technology, applied to either a turned surface or an SLA surface, on the mechanical robustness of osseointegration, benchmarked against the SLActive surface.

**Material and Methods:**

Ti discs (6.25‐mm‐diameter and 2‐mm‐thick) with three different surfaces were inserted on the proximal‐anterior part of the tibial plateau of adult Swedish loop rabbits: (I) turned surface modified with Ti‐Sr‐O (turned + Ti‐Sr‐O), (II) SLA surface modified with Ti‐Sr‐O (SLA + Ti‐Sr‐O), and (III) SLActive surface (SLActive). Following a healing period of 2 weeks and 4 weeks, the pull‐out (PO) force needed to detach the discs from the bone was assessed, as a surrogate of osseointegration.

**Results:**

The SLActive surface exhibited statistically significant higher median PO forces, compared with the SLA + Ti‐Sr‐O surfaces at both 2‐ and 4 weeks post‐op (*p* > .05). In this study, no single turned + Ti‐Sr‐O surface disk was integrated.

**Conclusions:**

The tested Ti‐Sr‐O technology failed to enhance osseointegration; however, this finding may be related to the inappropriateness of the rabbit tibia plateau model for assessing third‐generation implant surface technologies, due to the limited diffusion and clearance at the disk‐bone interface.

## 1 BACKGROUND

Surface modifications and functionalizations of titanium (Ti) implants draw the attention of continued research aiming for increasing the bio‐affinity to the hard tissue and accelerating the biological process of osseointegration (Fan et al., 2017; Mao‐Suan et al., 2015). Several surface functionalization routes have been developed, primarily based on topographical and chemical surface modifications (2nd generation surface technologies) in the macro‐, micro‐, and nano‐meter range, (Smeets et al., 2016) including grit‐blasting, acid‐etching, (Choi & Park, 2018) laser ablation, anodic oxidation, (El‐Banna et al., 2020) and hydrophilization under N2 protection/storage in liquid (Offermanns, Andersen, Sillassen, et al., 2018). Some of these surface technologies have already been shown to increase the production of factors involved in bone healing and remodeling and provide accelerated osseointegration (Shanbhag et al., 2015; Stavropoulos et al., 2021). Nevertheless, 3rd generation implant surface technologies resulting in bioactive surfaces (e.g. with the implementation of osteopromoting ions such as Zn, Si, Mg, B, and Ca) have gained increased attention in the pursuit of designing surfaces with enhanced osteogenic properties (El‐Banna et al., 2020; Su et al., 2019).

Sr (Strontium), an alkaline earth metal and chemical element with atomic number 38, possesses some properties similar to Ca and hence has the potential to be included in the mineral phase of bone. Sr implements both anabolic and catabolic effects on osteoblasts and osteoclasts and has been utilized previously in the treatment of osteoporosis (O'Donnell et al., 2006; Stevenson et al., 2007). These effects, is believed, are mediated by upregulation of runt‐related transcriptional factor 2 (RUNX2), activation of mitogen‐activated protein kinase phosphorylation and canonical and noncanonical Wnt/β‐catenin signaling (Borciani et al., 2022; Yang et al., 2011); while osteoclastogenesis seems regulated through the suppression of NF‐κB signal transduction (Yamaguchi & Neale Weitzmann, 2012). Systemic administration of Sr ranelate, in the form of Sr, appears to somehow improve osseointegration and peri‐implant bone quality in animals, (Scardueli et al., 2018) and to enhance bone regeneration in augmented defects in both healthy and osteoporotic rats (Mardas et al., 2021). Considering local application, a preclinical in vivo study showed that grafting of a Sr‐loaded bone material significantly increased bone formation (BF), in comparison with nonloaded controls (Aroni et al., 2019). Furthermore, a number of in vitro and in vivo studies on Sr ions incorporated into Ti implants have demonstrated positive effects on the osteogenic differentiation of bone marrow mesenchymal stem cells (rBMSCs). Sr has recently been found to be associated with osteoimmunomodulation leading to macrophage polarization towards the M2 type, rather than M1 type (Xu, Xie, et al., 2021). At the implant‐bone interface, sustained release of Sr has been achieved with a nanostructured Ti‐based surface coating comprising Sr and oxygen (O), by using magnetron sputtering. This Ti‐Sr‐O technology has shown favorable results in previous studies, in terms of enhanced osseointegration (Andersen et al., 2013; Offermanns et al., 2016; Offermanns, Andersen, Sillassen, et al., 2018; Offermanns, Steinmassl, et al., 2018).

Specifically, findings from small animal models (i.e., rats [Andersen et al., 2013; Offermanns et al., 2016] and rabbits [Offermanns, Andersen, Riede, et al., 2018; Offermanns, Andersen, Sillassen, et al., 2018; Offermanns, Steinmassl, et al., 2018]) have suggested that the Ti‐Sr‐O coating contributed positively to the osseointegration process and that more bone was formed around the Ti‐Sr‐O modified implants, relative to implants with a hydophilic sandblasted and acid‐etched surface (i.e., SLActive). More recently, micro‐/nano‐rough Ti surfaces incorporating Sr have been reported to exhibit advantages on osseointegration and early new BF compared to SLA and resorbable blasting media treated surfaces in the rabbit femur/tibial epiphysis model (Wu et al., 2020). Nevertheless, the aforementioned studies have been based on histological evaluation and no information exists demonstrating the relative mechanical robustness of osseointegration with this Ti‐Sr‐O technology. To determine the strength of the bone‐to‐implant attachment, mechanical‐based readouts (e.g., torque‐out and/or perpendicular pull‐out [PO]) are necessary.

Thus, the aim of the current study was to investigate the impact of the Ti‐Sr‐O technology, applied to either a turned surface or an SLA surface, on the mechanical robustness of osseointegration at early stages of healing, benchmarked against the SLActive surface. The primary hypothesis of the study was that the mean mechanical PO force for the Ti‐Sr‐O coated SLA (Institut Straumann AG) surface would be superior to that found for the SLActive surface, following a healing period of 2 and 4 weeks.

## 2 MATERIALS AND METHODS

### 2.1 Material preparation and surface treatments

The Ti disks used in the current study were prepared as Ø6.25 × 2.00 mm turned blanks, via a turning process, using the RxD alloy (Institut Straumann AG). Two‐thirds of the disks were then further modified to produce the SLA surface and the SLActive surface (Institut Straumann AG), using standard manufacturing processing parameters. Subsequently, the Ti‐Sr‐O surface was implemented to both turned and SLA‐modified blanks using previously described process and methods of characterization (Andersen et al., 2013; Sillassen et al., 2014). In short, samples were mounted in an industrial‐scale magnetron sputtering setup (CemeCon AG, Wuerselen, Germany). The coating process was allowed to run until a 1.8 µm thick coating had been deposited. Subsequently, process control was performed (data not shown) by assessing coating thickness and morphology by Scanning Electron Microscopy (SEM, Nova 600; FEI Company); Sr release was assessed through washout and analysis using the method of Inductively Coupled Plasma Optical Emission Spectroscopy (ICP‐OES, (AMETEK Spectro Arcos, AMETEK). Thus, three types of surface modifications were tested in the current study: The Ti‐Sr‐O surface applied to either a (I) turned surface (turned + Ti‐Sr‐O) or (II) to an SLA surface (SLA + Ti‐Sr‐O) and (III) the SLActive surface acting as the control (SLActive).

### 2.2 Animals and surgery

The present in vivo study was conducted at the Biomedical Center, Lund University, Lund, Sweden. All experiments were carried out in accordance with the Swedish Animal Protection Law and approved by the Animal Ethics Committee at Lund University (ethical approval number M 138‐14). The study is reported according to the ARRIVE guidelines for the related substances (Berglundh et al., 2012).

A total of 25, 6‐month‐old, Swedish loop rabbits of both sexes (Christer Månsson), weighing 3.3–4.3 kg, were used for the present study. The animals were kept in interconnecting single cages under standard humidity (40–70%) and temperature (20–24°C) conditions in 12:12 day–night cycles and had access to water ad libitum and standard laboratory animal diet (RABMA® [Rabbit Maintenance], 4 mm pelleted #803550, Special Diet Services). The animals were acclimatized for at least 7 days in test conditions after the health assessment. All animals did not show any visible signs of illness when included in the current investigation.

**Figure 1:**
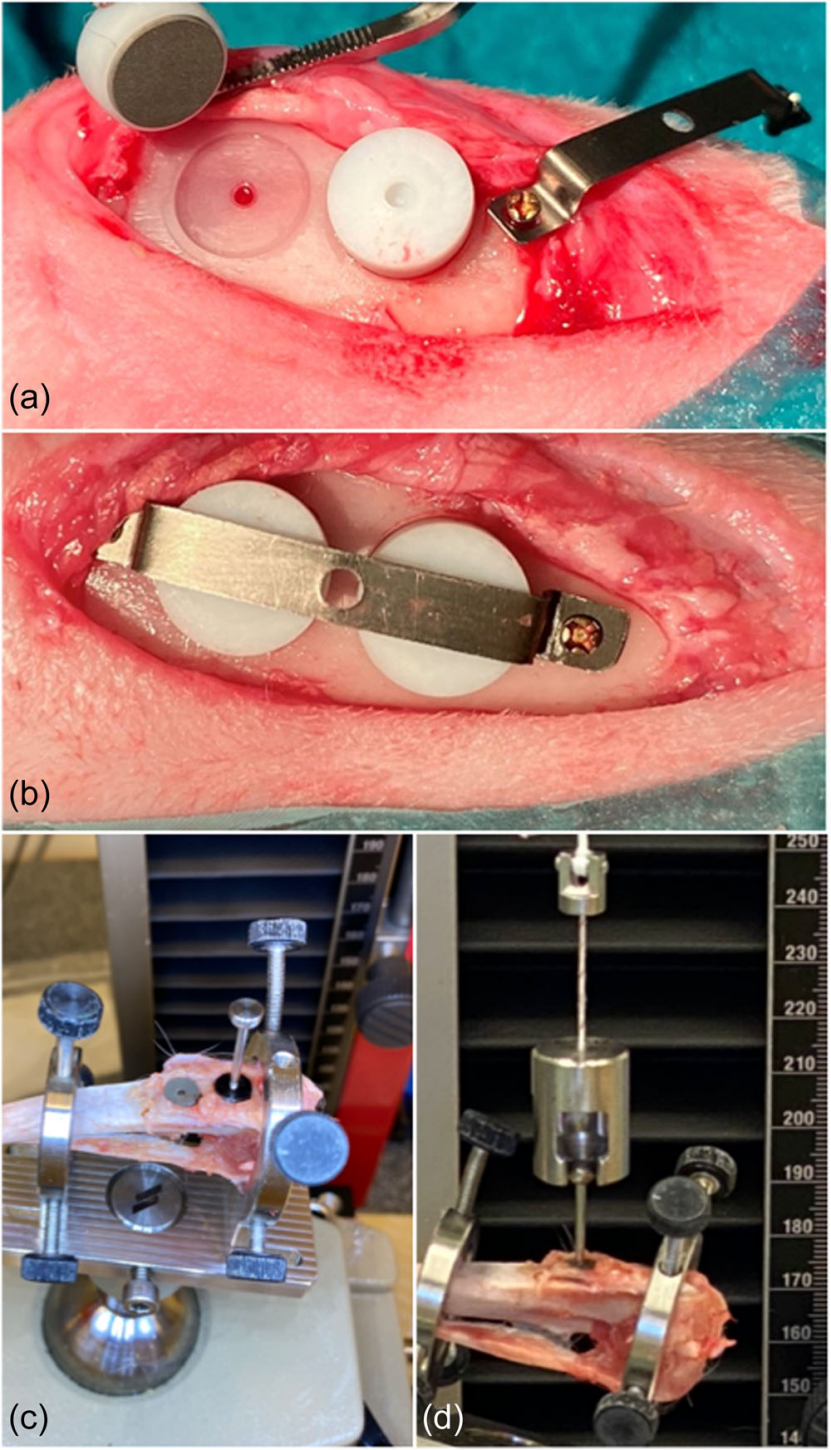
The disks were placed on a bed prepared on the tibia plateau and covered with a teflon cap (a) and stabilized with an overlying metal band and mini‐screws (b). For pull‐out testing, a stylus with a rounded head was connected to the disks (c) and this, in turn, via a cylindrical holder to a special pull‐out testing device with a load‐cell (d).

All surgical procedures were conducted under general anesthesia as previously described (Pippenger et al., 2019). Briefly, anesthesia was provided by means of intravenous injections of metetomidin (Dormitor Vet, Orion Pharma, Espoo, Finland, 1 mg/mL, 0.15 mL/kgbw) and ketamin (Ketalar Vet, Pfizer AB, Sollentuna, Sweden, 50 mg/mL, 0.35 mL/kgbw). In each tibia, the surgical areas were injected with 0.9 mL of lidocain/epinephrine solution per site (Xylocain Dental adrenalin 20 mg/mL + 12.5 mg/mL, Astra AB) to achieve local anesthesia. The surgical site was depilated and washed with soft soap and ethanol. Under aseptic conditions, an incision was performed through all soft tissue layers on the proximal‐anterior part of the tibiae. Following the elevation of periosteum and stabilization by a self‐retaining retractor, four guide holes were prepared with a 1.0‐mm‐diameter twist drill (Medartis, AG) by means of a drill guide to provide standardized and correct positioning of two disks per tibia. Then, a bed was prepared for each disk under copious physiological saline solution irrigation, to allow placing the disk flat on the tibial cortical bone, utilizing a low‐speed rotating dental implant hand piece with a custom‐made 7.05‐mm‐diameter bur. A polytetrafluoroethleyene (PTFE) cap was then placed over each disk to prevent vertical bone growth on the side of the disk and was stabilized with a 0.25 mm titanium band mounted across both cups and retained using two 1.2 × 3 mm titanium screws (Medartis, AG) (Figure 1). Eventually, the tissues were sutured in layers using a resorbable suture (Vicryl 4‐0, FS2, Ethicon, Inc.).

With 25 animals, the current split‐leg design provides 100 implantation sites. Block randomization was followed to have a balanced number of disks accounting for positioning (proximal vs. distal) and leg (right vs. left) and to allow a minimum sample size of 9 (*n* = 9) per time point (2 and 4 weeks); specifically, for the SLActive group 10 and 9 disks, for the SLA + Ti‐Sr‐O group 11 and 10 disks, and for the turned + Ti‐Sr‐O group 11 and 10 disks were allocated to the 2‐ and 4‐week observation time, respectively. The remaining sites received disks of different technologies, not reported herein. After the operation, the rabbits were brought back into their cages and analgesia (Temgesic, Schering‐Plough AB, 0.3 mg/mL) was provided for 3 days. The rabbits were kept in the animal facility without transportation during the healing phase.

Two and 4 weeks after the implantation procedures, the respective rabbits were killed by an overdose of pentobarbital (Pentobarbitalnatrium, 60 mg/mL; VET ATL, Apoteket). The legs were cut 5 cm below the knee joint and were wrapped in gauze soaked with a 0.9% saline solution, before analysis, to avoid drying.

### 2.3 Biomechanical PO measurements

The procedures for the PO analysis were performed as previously described (Rønold & Ellingsen, 2002). Briefly, after incising the skin on the tibial bone, the titanium band covering the PTFE cup implants was exposed and removed. The PTFE caps were removed by drilling a hole with a hollow needle and then applying pressurized air. Then, the tibial bones were mounted in the PO testing device (Zwick Roell Z 2.5; Zwick GmbH, Ulm) fitted with a calibrated load‐cell of 250 N with a cross‐head speed of 1.0 mm/min, and adjusted to ensure alignment with the load cell, using a level tube. To reduce the effect of shear forces, an approximately 100 mm long thread is connected between the load‐cell and the screw engaging the disk. The load was applied until the disc loosened and recorded on the load versus time graph.

### 2.4 Statistical analysis

Mechanical PO data were summarized as mean values ± sd, and median and interquartile range (IQR) were plotted in box plots and checked for outliers. The normality data were evaluated by using The Shapiro‐Wilk test and Levene's test was used for the homogeneity of variances for each group. To analyze the data of the groups at the same time point, the Kruskall–Wallis test was used for multiple comparisons while, the Mann–Whitney *U* test was performed for pairwise comparisons. *p* < .05 was defined as statistically significant. All statistical analyses were done by using IBM SPSS Statistics for Windows, (Version 23.0.) (IBM Corp.).

### 2.5 Ethics statement

2.5.1

All experiments were carried out in accordance with the Swedish Animal Protection Law under the ethical approval from the Animal Ethics Committee at Lund University (ID number: M 138‐14).

## 3 RESULTS

All animals completed the 2‐, and 4‐week observation time periods. During the healing period, mild edema/hematoma was observed in all operation areas except for two right legs in two animals, that exhibited signs of dehiscence at the surgical site, and were treated by resuturing. Complications, that is, infection, allergic reaction, and implant loss, were not noticed.

**Figure 2:**
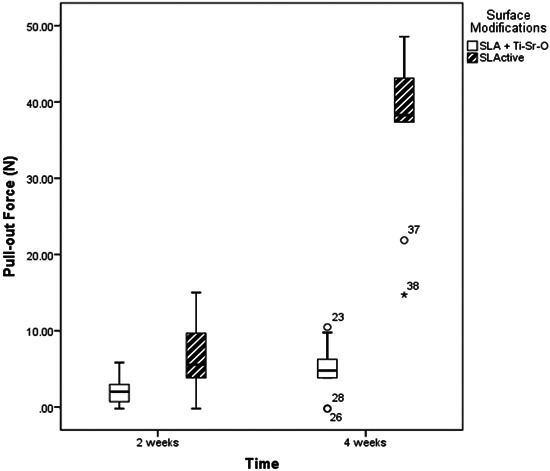
Comparison of the median pull‐out forces (N) of the implant surface modifications at 2 and 4 weeks postoperatively. In both healing time periods, no single Turned + Ti‐Sr‐O surface disc was integrated and therefore this group could not have a pull‐out value and could not be represented on the graph as surface modification.

All disks of the SLActive and SLA + Ti‐Sr‐O group integrated. From the obtained data, it was found that the SLActive surface exhibited the highest mean PO force. Specifically, at the 2 weeks healing time, the SLActive surface had a mean (median [IQR]) PO force of 7.33 ± 5.24 N (7 [3−11.93]), which was significantly superior to that of the SLA + Ti‐Sr‐O surface (2.31 ± 1.95 N; 2.2 [0−3.7]) (*p* = .023). Similarly, at the 4 weeks healing period, there was a significant difference between SLActive and SLA + Ti‐Sr‐O surfaces in favor of the former (36.44 ± 11 N; 38.2 [29.6−43.45]) versus (5.06 ± 3.49 N; 4.95 [3−7.27]), respectively) (*p* < .001). At both healing time periods, no single turned + Ti‐Sr‐O surface disk was integrated (Figure 2).

## 4 DISCUSSION

The present preclinical in vivo study, using the rabbit tibia plateau model, aimed to investigate the potential of the Ti‐Sr‐O surface technology to enhance osseointegration when applied to either turned or SLA‐modified surfaces, by assessing mechanical PO forces, a reliable surrogate measure for osseointegration (Brånemark et al., 1997). The findings of the study showed that the Ti‐Sr‐O surface technology, both when applied on a turned or SLA surface, was significantly inferior to the SLActive surface, at a healing period of 2 and 4 weeks.

Over the last decades, efforts have focused on implant surface biofunctionalization, aiming at accelerating and improving the osseointegration process and the long‐term survival of implants (El‐Banna et al., 2020; Offermanns, Andersen, Sillassen, et al., 2018; Shanbhag et al., 2015). Morphological and hydrophilic surface modifications, i.e., second generation technologies, indeed improve BF and osseointegration; third‐generation surfaces technologies, however, based on modification of the physico‐chemical properties of the implant surface itself, and so forth, addition of calcium polyphosphates (Ca‐P), (Hatt et al., 2019) Mg, (Lee et al., 2020) and Sr (Lin et al., 2019; Offermanns et al. 2016; Offermanns, Andersen, Sillassen, et al., 2018; Offermanns, Steinmassl, et al., 2018; Xu, Zhang, et al., 2021) seem to have a more pronounced potential in promoting osseointegration and host‐to‐implant response. In particular, the Ti‐Sr‐O technology tested herein has been found to improve osseointegration in several preclinical in vivo studies (Andersen et al., 2013; Offermanns et al., 2016; Offermanns, Andersen, Riede, et al., 2018; Offermanns, Andersen, Sillassen, et al., 2018; Offermanns, Steinmassl et al., 2018). For example, a nanopatterned Ti‐Sr‐O functionalized titanium surface was compared to an SLActive surface regarding new BF% and bone‐to‐implant contact (BIC%) in a rabbit femoral condyle model (Offermanns, Andersen, Sillassen, et al., 2018). This Ti‐Sr‐O surface technology has achieved larger amount of new BIC%, however with no statistical significance, in comparison to SLActive implants at 2 weeks of healing (Offermanns, Andersen, Sillassen, et al., 2018). Similarly, implants with Sr incorporated on an SLA surface, with a different approach than the current Ti‐Sr‐O technology, showed improved osseointegration compared to control SLA implants when placed in the rabbit proximal tibiae and femoral condyles (Fan et al., 2017). The contradicting results obtained in the current study appear at first glance very surprising, but on second thought seem related to the specific model herein. In the tibia plateau model, the disks are placed on equal‐sized beds prepared on the tibia plateau and thus the entire titanium surface is in very close contact with the bone cortex (i.e., pure cortical bone model) (Wennerberg & Albrektsson, 2010). It may be assumed that due to this tight contact and the characteristics of the cortical bone, there was inadequate transport of Sr away from the site of implantation, which in turn led to very high concentration of Sr ions and/or very high pH at the disk‐bone interface in the turned + Ti‐Sr‐O group.

Indeed, from a microstructural point of view, cortical bone is characterized by a lower degree of vascularization and lower water content, in general, and thus it possesses decreased rates of Gaussian diffusion (i.e., when the diffusion of water is not affected by physical constraints) compared to trabecular bone and bone marrow (Aoki et al., 2016; De Santis et al., 2010; Dieckmeyer et al., 2017; Fernandez‐Seara et al., 2002). The water content for mature cortical bone from the rabbit tibia is about 10%, (Fernández‐Seara et al., 2002) whereas it has been found as high as 40% for hematopoietic bone marrow (Aoki et al., 2016). The apparent diffusion coefficient of “unbound” water, i.e. water that is free to move around in the extracellular matrix and structures of the various tissues, is for mature cortical bone (Fernández‐Seara et al., 2002) 10−100 times slower compared with that in bone marrow (De Santis et al., 2010; Dieckmeyer et al., 2017). Thus, considering these values, the ability of cortical bone to facilitate transport of low‐molecular weight degradation products can be estimated to be between 40 and 400 times lower than that found for bone marrow. Sr is a water‐soluble ion and thus its diffusion depends on the diffusion of water at the site of implantation. In an attempt to calculate the concentration of Sr at the disk‐bone‐interface, in the tibia plateau model herein, it was assumed a gap distance of 1 µm between the disc and the bone surface; and that this gap is completely occupied by fluid and there is no net transport of Sr away from the gap at the very early healing phase. With a disk surface area of 0.4 cm^2^, the gap volume would be 4 × 10^−5^ mL, and with the current Ti‐Sr‐O technology the release from the turned surface is approximately 30 µg/cm^2^ over a 14 days period (Offermanns, Andersen, Sillassen, et al., 2018); this translates to a release of Sr—in the form of Sr(OH)_2_—at a concentration of 0.3 g/mL. Based on the solubility of Sr(OH)_2_, being 0.15 mol/L at 40°C, (Lambert & Clever, 2013) and assuming that the solubility in the fluid in the gap is comparable to that found in pure water, the Sr concentration at the disk‐bone interface may reach 13 mg/mL, while the pH may reach 13.5, a short time after placement of the disks. Considering the relatively low rate of diffusion in cortical bone, as mentioned above, coupled with the reservoir of Sr in the Ti‐Sr‐O implant surface, it is likely that the too high Sr concentration and the elevated pH persist for a significant period of time at the disk‐bone interface of the turned + Ti‐Sr‐O group; it is likely that the Sr(OH)_2_ concentration in the interface fluid volume is replenished as quickly as its concentration is decreased by diffusion. In this context, it has been reported that the upper limit of pH supporting osteogenesis is at 8.4, (Galow et al., 2017) while high Sr concentration may interfere with osteoblastic activity and mineralization (Ammann et al., 2004; Bonnelye et al., 2008; Fan et al., 2017; Sila‐Asna et al., 2007). For instance, 72 µg/mL Sr in osteoblastic cell cultures inhibited differentiation (Sila‐Asna et al., 2007) and a concentration of 20 or 100 µg/mL was shown to compromise mineralization (Verberckmoes et al., 2003). In perspective, the finding that the SLA + Ti‐Sr‐O disks achieved some degree of osseointegration implies that the Sr concentration and/or pH at the disk‐bone interface in the SLA + Ti‐Sr‐O disks was compatible with osseointegration and might be explained by unpublished data showing that Sr release is about 25% less, compared with the turned + Ti‐Sr‐O discs. Furthermore, it is likely that the SLA disks were more readily covered with—even limited in this model—blood compared with the turned + Ti‐Sr‐O discs. It is reasonable to assume that this blood coverage, and the corresponding high degree of protein adsorption at the surface of the implant, negatively affects the release kinetics of Sr from the surface of the disk, thus reducing the Sr concentration and pH at the disk‐bone interface. In this context, a 2‐ and 4‐week healing interval was chosen herein to assess the possible impact of the Ti‐Sr‐O technology in enhancing osseointegration at early stages of healing, since a major part of bone remodeling—and thus osseointegration—is considered completed in the rabbit ca. 6 weeks after bone trauma. Nevertheless, prolonging the healing time, it would not have significantly changed the conclusions, even if the SLA+Ti‐Sr‐O disks had caught up with the SLActive ones. Furthermore, from a strict methodology point of view, the SLA + Ti‐Sr‐O surface should have been compared to the SLA surface and the turned + Ti‐Sr‐O to the turned surface. However, the SLActive surface, and not the SLA or the turned Ti surfaces, was used as the benchmark herein, since—as discussed above—it is a surface with proven capacity to accelerate osseointegration.

## 5 CONCLUSION

The tested Ti‐Sr‐O technology failed to enhance osseointegration; however, this finding may be related to the inappropriateness of the rabbit tibia plateau model for assessing third‐generation implant surface technologies, due to the limited diffusion and clearance at the disk‐bone interface.

## AUTHOR CONTRIBUTIONS

**Sila Cagri Isler:** Manuscript drafting; data interpretation. **Benjamin Bellon:** Biomechanical testing; data curation; manuscript reviewing. **Morten Foss:** Surface characterization; data interpretation; manuscript reviewing. **Benjamin Pippenger:** Biomechanical testing; data curation; manuscript reviewing. **Andreas Stavropoulos:** Surgeries; data interpretation; manuscript drafting. **Ole Zoffmann Andersen:** Surgeries; data interpretation; manuscript drafting.

## CONFLICT OF INTEREST STATEMENT

B. B., B. P., and O. Z. A. are employed by Institut Straumann. The remaining authors declare no conflict of interest.

## Data Availability

The information supporting the findings of the current study are available from the authors in case of reasonable request.
